# Surgical ventricular restoration with CABG with or without mitral repair: is it an ideal surgery?

**DOI:** 10.1186/1749-8090-8-S1-O190

**Published:** 2013-09-11

**Authors:** Y Menaissy

**Affiliations:** 1 Department of Cardiothoracic Surgery, Cairo University, Cairo, Egypt

## Background

Surgical ventricular restoration by means of the Dor procedure is a surgical option in patients with coronary artery disease, post- infarction left ventricular aneurysm or ischemic dilated cardiomyopathy with or without mitral regurgitation. The aim of this study was to evaluate our 8 year clinical experience of this procedure.

## Methods

From April 2005 to April 2013, surgical ventricular restoration was performed in 48 patients (36 males), mean age 57 (41-74) years. All patients presented with angina and/or heart failure and/or ventricular tachycardia. Postinfarction left ventricular aneurysm was present in all 48 patients and ischemic dilated cardiomyopathy with a large akinetic left ventricle in 8. The preoperative left ventricular ejection fraction was 32+/-9 (19-43) %. Multi-vessel disease was present in 18 patients. Ventricular tachycardia was diagnosed in 10 patients (spontaneous VT in 2). Mitral regurgitation more than grade I was found in 18 patients. The mean EuroSCORE was 7.3+/-2.6 (4-17).

## Results

All patients underwent the Dor procedure, which included a non-guided endocardectomy to exclude the aneurysm and treat ventricular tachycardia. Coronary artery bypass grafting was performed in all 48 patients and a mitral valve repair was performed in 18. Intra-aortic balloon pumping was used preoperatively in 7 patients and postoperatively in 6 cases and all 48 patients needed inotropic support for more than 24 h. Hospital mortality was 8/48 (16.6%).

## Conclusions

The Dor ventricular restoration is a good surgical option for treatment of postinfarction left ventricular aneurysm and mitral repair should be done if the regurgitation is more than mild. Eight years results are good compared to the patients EuroSCORE.

